# Reduced expression of PD-L1 in autoimmune thyroiditis attenuate trophoblast invasion through ERK/MMP pathway

**DOI:** 10.1186/s12958-019-0536-1

**Published:** 2019-10-27

**Authors:** Mengya Chen, Nduwimana Gilbert, Haixia Liu

**Affiliations:** Department of Endocrinology and Metabolism, The Second Affiliated Hospital of Dalian Medical University, 467 Zhongshan Road, Dalian, 116027 Liaoning China

**Keywords:** PD-1/PD-L1, AIT, Placenta, Invasion, ERK1/2

## Abstract

**Background:**

Autoimmune thyroiditis (AIT) with euthyroid is associated with miscarriage. But the exact mechanism remains unclear. Studies have shown that the programmed cell death-1 (PD-1)/programmed cell death -ligand 1 (PD-L1) pathway is essential for normal pregnancy. However, the expression of PD-L1 in gestational trophoblasts in mice with autoimmune thyroiditis and the mechanisms leading to miscarriage have not been fully investigated.

**Methods:**

Immunofluorescence and Western blot were used to detect the expression of PD-L1, p-ERK, MMP-2 and MMP-9 in embryonic trophoblast cells of pregnant mice with AIT. The expression of PD-L1 in HTR-8/SVneo cells were silenced, and the expression of PD-L1, MMP-2, MMP-9, ERK and p-ERK1/2 was detected by Western blot analyses and immunofluorescence assays. Invasive assays were performed in PD-L1 silenced HTR-8/SVneo cells using a Transwell chamber.

**Results:**

Compared with normal pregnancy, the expression of PD-L1, ERK, p-ERK, MMP-2 and MMP-9 in embryonic trophoblast cells was significantly lower in pregnant mice with AIT. Compared with the negative control (NC) group (cells transfected with negative control siRNA), phosphorylation of MMP-2, MMP-9 and P-ERK1/2 proteins was significantly reduced in HTR-8/SVneo cells transfected with PD-L1 siRNA, and the number of cells penetrating the membrane was reduced.

**Conclusion:**

AIT inhibits ERK/MMP-2 and MMP-9 pathways through PD-L1 reduction, attenuates embryonic trophoblast invasion and ultimalely induces miscarriage ultimately.

## Introduction

A lot of evidence suggests that the most common and serious adverse pregnancy outcomes caused by AIT with euthyroid are miscarriages, especially repeated abortions [1, 2]. However, the specific mechanism is still unclear. The invasive behavior of extravillous trophoblast cells (EVT) is an essential element in the formation and development of placenta and the successful completion of pregnancy [3, 4]. EVT cells originate from cytotrophoblastic (CTB) cells and then invade the upper third of the aponeurosis and myometrium while reconstructing the associated spiral arteries [5]. The invasive ability of EVT cells is strictly regulated by various growth and regulatory factors in the endometrial microenvironment of the entire pregnancy, mainly the decidua [6]. This regulation is carried out in a strict space and time mode, and the destruction of such regulations may lead to adverse consequences [3, 7].

PD-1/PD-L signaling pathway is a negative costimulatory pathway found in recent years [8]. PD-1 is mainly expressed on the surface of activated T cells, while PD-L1 is mainly expressed in antigen-presenting cells and immunologically exempt sites (such as placenta). Holets et al. [9] found that PD-L1 is expressed on all trophoblast cells in human placenta. The PD-L1 protein of tumor cells binds to the PD-1 receptor on T cells, T cells cannot recognize tumor cells, and tumor cells open immune escape, which gives the tumor cells strong invasiveness [10, 11]. Studies have shown that extracellular signal-regulated kinase (ERK) signaling is mediated by PD-L1, which activates ERK signaling through PD-L1 to confer tumor invasiveness [12, 13]. The invasion of trophoblast cells is similar to that of tumor cells [3]. In addition, ERK1/2 phosphorylation has been shown to be involved in the regulation of MMP-2/− 9 expression [14]. The trophoblast invasion ability is closely related to the expression of MMP-2/− 9, because MMP-2/− 9 is the main enzyme that degrades the basement membrane, and the extracellular degradation matrix (ECM) and basement membrane are the initial processes to promote cell invasion [15, 16]. Studies have shown that the ERK signal transduction pathway regulates the invasion of trophoblasts by activating cell adhesion molecules and myosin, up-regulating the expression of MMP-9 [17]. The study found that there were MMP-2 and MMP-9 synthesis in the decidual cells of the pseudopregnant mice on days 6–8 [18]. In vitro experiments showed that the mRNA and protein of MMP-2 and MMP-9 were elevated in trophoblast cells in early pregnancy [19]. Based on the above evidence, we hypothesized that AIT due to the decrease of PD-L1 content in embryonic trophoblast cells, which then affects the expression of MMP-2/− 9 through ERK signaling pathway, and reduce the invasiveness of trophoblast cells to induce abortion.

## Materials and methods

### Immunisation protocols

AIT modelling of CBA/J mice with thyroglobulin using previously reported methods [20]. In order to induce autoimmune thyroiditis, CBA/J mice were first immunised with mTg (100 μg/mouse) in complete Freund’s adjuvant (Sigma, F5881) at 5 weeks of age and were then challenged with mTg (100 μg/mouse) in incomplete Freund’s adjuvant (Sigma, F5506) at 7 weeks of age. The same dose of phosphate buffered saline (PBS) instead of mTg was used to immunize the control group mice, and the other methods were the same as those of the mTg group. CBA/J (Beijing HFK Bioscience Co) AIT pregnant mice were sacrificed by cervical dislocation at 13.5th day of gestation, and placental tissue was isolated by laparotomy.

### Thyroid function tests

TT_4_ (Signalway Antibody, EK18886) TSH (Elabscience Biotechnology Co, Ltd) and anti-Tg antibody (Elabscience Biotechnology Co, Ltd) were determined by ELISA. All samples were measured twice, and specific experimental steps were performed in accordance with the kit specifications.

### Immunohistochemistry

The embedded placental tissue sections were treated using standard immunohistochemical techniques. Antibodies used in immunohistochemistry experiments included an anti-PD-L1 Ab (1:800 dilution; Proteintech, 66,248–1-Ig), anti- p-ERK Ab (1:300 dilution; CST, Thr202/Tyr204, mAb #4370), anti-MMP-2 (1:200 dilution; Proteintech, 10,373–2-AP) and anti- MMP-9 Ab (1:200 dilution; Proteintech, 10,375–2-AP). Immunoreactivity was evaluated independently by two investigators who were blinded to experimental protocol according to the intensity and extent of staining. Immunohistochemistry images were obtained with confocal microscopy (Leica DM4000B) and immunoreactivity using at least 3 random microscopes field of view. The experimental results were analyzed using Image-Pro Plus software (version 6.0; Media Cybernetics).

### Cell culture

HTR-8/SVneo cells (EK-Bioscie, Shanghai, CHN) were cultured in RPMI-1640 medium (HyClone, US) containing 10% fetal bovine serum (FBS, AusGeneX, AUS), 1% penicillin-Streptococcus bismuth antibody (HyClone, US), and incubated under 5% CO_2_ at 37 °C.

### siRNA transfection and gene silencing

HTR-8/SVneo cells were classified into a control group, a small interfering negative control (NC) group (cells transfected with negative control siRNA) and two different small interfering RNAs groups (cells transfected with PD-L1 siRNA1 and siRNA2). The PD-L1 siRNAs and negative control siRNA used in the present study were provided by Shanghai GenePharma Co., Ltd. (Shanghai, China). The transfection of cells was performed with Lipofectamin2000 (Invitrogen, Carlsbad, CA) for a final concentration of 50 nM siRNA/well, according to the manufacturer’s protocol. Cells were harvested 48 h post-transfection for further analyses. The inhibition efficiency was identified by western blot. The sequence of PD-L1 siRNA1: sense: 5′-GCC GAA GUC AUC UGG ACA ATT-3′, antisense: 5′-UUG UCC AGA UGA CUU CGG CTT-3′. The sequence of PD-L1 siRNA2: sense: 5′-GAA GCA AAG UGA UAC ACA UTT-3′, antisense: 5′-AUG UGU AUC ACU UUG CUU CTT-3′. The sequence of NC siRNA: sense: 5′-UUC UCC GAA CGU GUC ACG UTT-3′, antisense: 5′-ACG UGA CAC GUU CGG AGA ATT-3′.

### Western blot analysis

Proteins were cleaved from CBA/J mouse placenta and HTR-8/SVneo cells for Western blot analysis. Standard Western blotting techniques were used to determine protein expression; 10 μg of protein was separated by 10% SDS-PAGE, transferred to a polyvinylidene fluoride membrane, and treated with anti-PD-L1 (1:2000 dilution; Proteintech, 66,248–1-Ig), anti- p-ERK Ab (1:2000 dilution; Cell Signaling, Thr202/Tyr204, mAb #4370), anti-MMP2 (1:2000 dilution; Proteintech, 10,373–2-AP), anti- MMP9 Ab (1:2000 dilution; Proteintech, 10,375–2-AP) and anti-GAPDH (1:2000 dilution; Cell Signaling) Ab overnight at 4 °C in a refrigerator and then with the second antibody (1:2000 dilution; Cell Signaling) it was incubated for 90 min. The signal was detected in an enhanced ECL system (GE/Amersham).

### Immunofluorescence staining and analysis

The cells cultured on the cell slides were washed three times with phosphate buffered saline (PBS), fixed with PBS containing 4% paraformaldehyde for 10 min, and then incubated in Triton X-100-containing PBS for room temperature permeabilization. After 10 min, the cells were washed with PBS, we added PD-L1 (1:200 dilution; Proteintech, 66,248–1-Ig), MMP-2 (1:200 dilution; Proteintech, 10,373–2-AP), MMP-9 (1:200 dilution; Proteintech, 10,375–2-AP), ERK1/2 (1:200 dilution; Cell Signaling) and phosphorylated ERK (1:200 dilution; Cell Signaling, Thr202/Tyr204, mAb #4370) primary antibody (1:200, Abcam) overnight at 4 °C, anti-rabbit fluorescent secondary antibody (1:50, Thermo) incubate at room temperature for 1 h, 1 μg/mL DAPI (Roche diagnostics) at 37 °C for 15 min, remove excess water, add fluorescent sealing solution, cover the coverslip, and observe under a laser confocal microscope (Olympus BX63).

### Cell invasion assay

HTR-8/SVneo cells in the control group, NC group, siRNA1 group and siRNA2 group were first incubated for 48 h. Cell invasion assays were performed using a transwell chamber (Corning) pre-coated with Matrigel (BD, USA). RPMI-1640 medium containing 15% FBS was added to 600 ml of the lower chamber. The cells were then washed with PBS and suspended in MEM. Two hundred milliliter of cell suspension (1×10^5^ cells/ml) was added to the upper chamber. After incubation at 37 °C for 36 h, the upper cells of the non-invasive membrane were wiped with a cotton swab. The filter was fixed in methanol and stained with 4 g/L crystal violet. The number of invading cells whose nuclei were stained purple was counted under microscope.

### Statistical analyses

Density values of proteins Bands obtained by Western Blot were detected by Image-pro Plus 6 software. All data were statistically analyzed using SPSS 20.0 or Graph Pad Prism6 software. The difference was statistically significant, *P* < 0.05.

## Results

### Information about the AIT pregnant mice

**Fig. 1:**
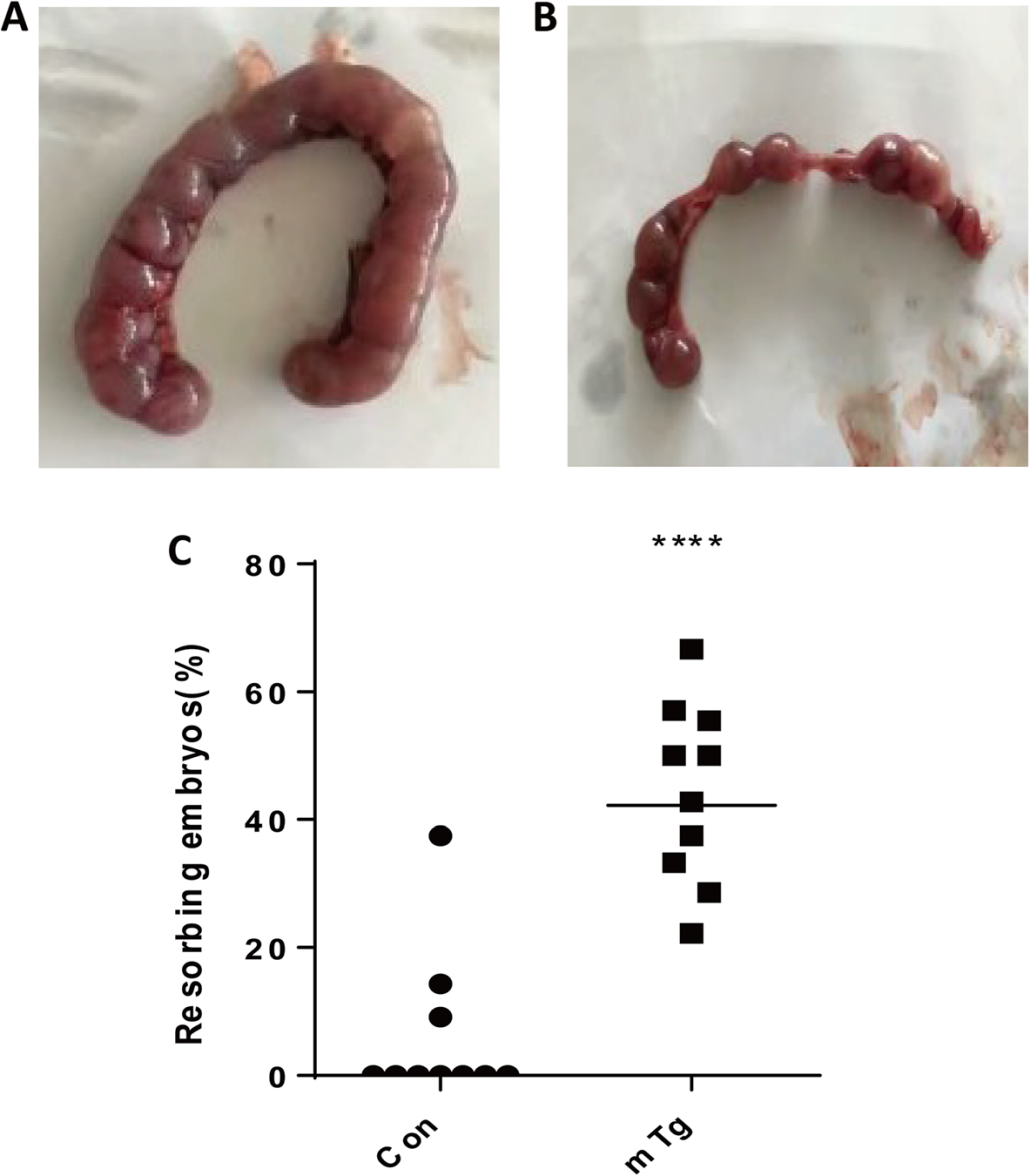
**a** Pregnancy embryo of the Con group. **b** Pregnancy embryo of the AIT mice. **c** Comparison of embryo absorption rates of each group

Building a murine fetal loss model of isolated positive maternal TgAb The first step of the experiment was to construct a TgAb-positive aborted animal model. No significant difference was found in the serum TT_4_ and TSH level after immunization with mTg (Table 1). The serum TgAb level in the mTg group was significantly higher than that in the con group (Table 2, *P* < 0.05). Embryo implantation rates were reduced in mTg group compared to control group (94.63% vs. 53.21%; *P* < 0.05. Fetal resorption rates were increased in mTg group compared to the control group (Fig. 1c) and the volume of embryos in the mTg group was significantly smaller than that of the control groups, as shown in Fig. 1a-b. This may be due to conditions such as ischemia, hemorrhage, and necrosis in the embryo.

**Table 1:** The comparison of the serum levels of TSH and TT4 ($\overline{x}$ ± S.E.M)

Groups	TSH (mIU/L)	TT_4_(μg/dL)
Con(*n* = 10)	0.3306 ± 0.03495	3.907 ± 0.4839
mTg(*n* = 10)	0.3165 ± 0.04059	3.595 ± 0.3682

**Table 2:** The comparison of the serum levels of TgAb ($\overline{x}$ ± S.E.M)

Groups	TgAb (μg/dL)
Con(*n* = 10)	1.805 ± 0.030
mTg(*n* = 10)	37.25 ± 2.800

### PD-L1, phosphorylated ERK, MMP-2 and MMP-9 expression in embryonic trophoblast cells of AIT pregnant mice

**Fig. 2:**
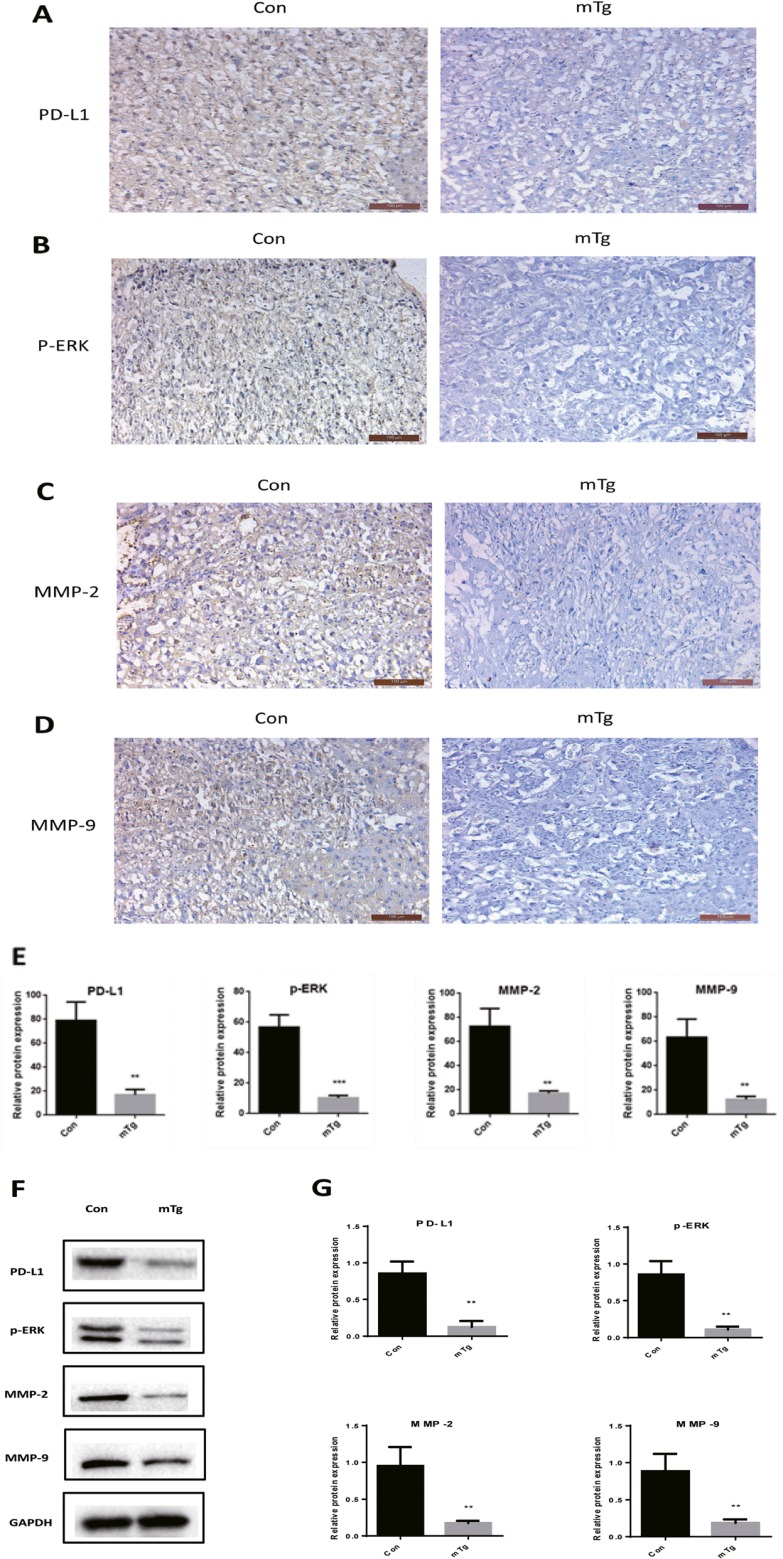
The expression of PD-L1 (**a**), p-ERK (**b**), MMP-2 (**c**) and MMP-9 (**d**) was detected by immunohistochemistry (× 100) in placental trophoblast cells of mouse placenta. **e** Statistical analysis of immunohistochemical results (** *P* < 0.01, *** *P* < 0.001). **f** Expression of PD-L1, p-ERK, MMP-2 and MMP-9 in placental trophoblast cells by Western blotting. **g** Statistical analysis of Western Blot (** *P* < 0.01)

The results of immunohistochemistry showed that, compared with the control group, the amount of PD-L1 (Fig. 2a) in the embryonic trophoblast cells of pregnant mice with AIT was significantly decreased. The amount of phosphorylated ERK (Fig. 2b), MMP-2 (Fig. 2c) and MMP-9 (Fig. 2d) was also significantly reduced.

To further compare the expressions of PD-L1 in the placenta, we used Western Blot to detect the PD-L1, t-ERK, p-ERK, MMP-2 and MMP-9 expressions in the placental tissue proteins of normal pregnant mice and AIT pregnant mice. The results showed that the PD-L1, p-ERK, MMP-2 and MMP-9 expressions in placental tissue proteins of AIT pregnant mice was significantly reduced (Fig. 2e).

### Silencing of PD-L1 reduced the levels of phosphorylated ERK, MMP-2 and MMP-9 of HTR-8/SVneo cells

**Fig. 3:**
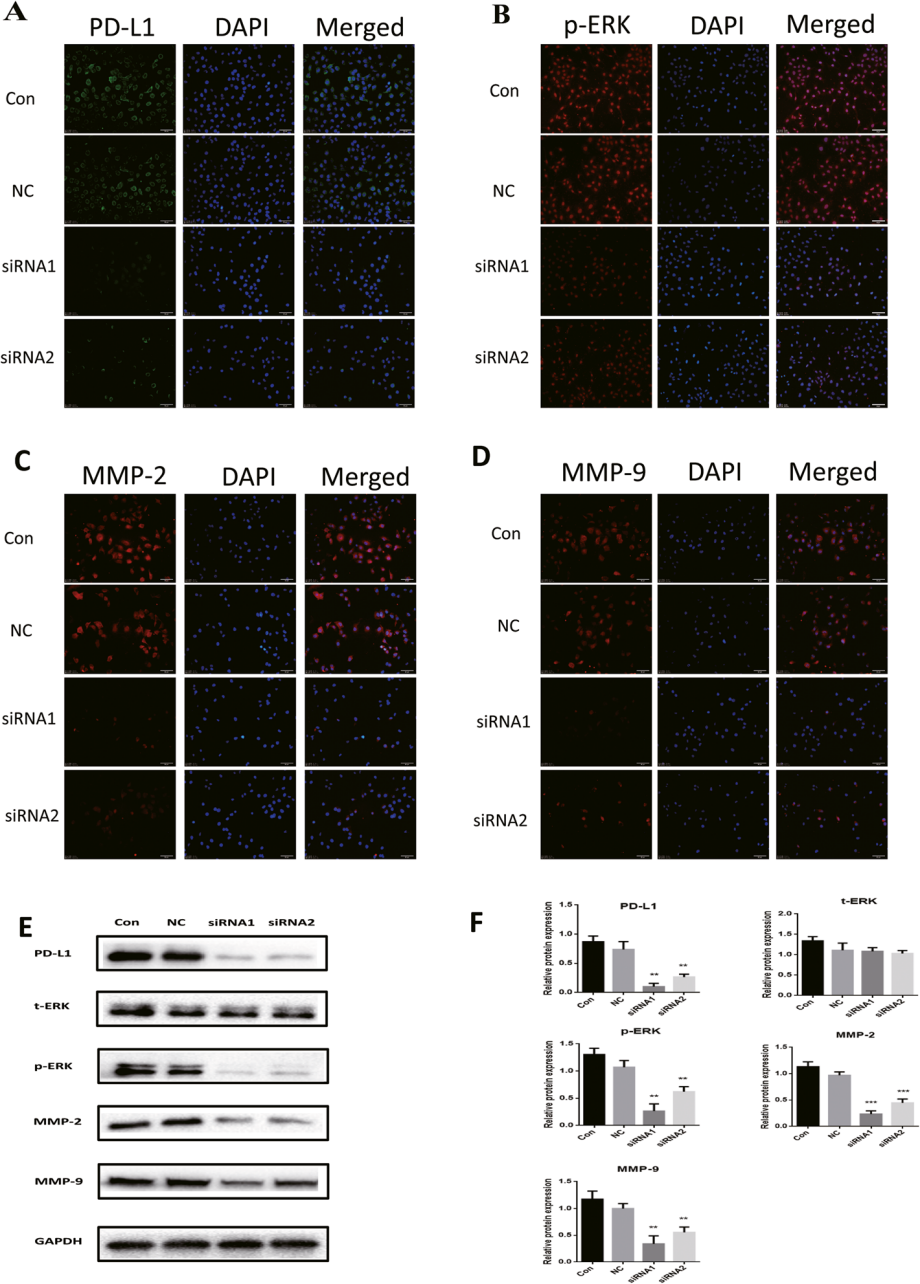
Effective knockdown of PD-L1 by siRNA in HTR-8/SVneo cells. HTR-8/SVneo cells were pre-incubated with PD-L1 siRNA for 48 h, and the levels of PD-L1 (**a**), p-ERK (**b**), MMP-2 (**c**) and MMP-9 (**d**) in NC and siRNA groups were detected by immunofluorescence; **e** The protein expression of PD-L1, MMP-2, MMP-9, ERK1/2 and pERK1/2 were evaluated by western blot in transfected cells. **f** Statistical analysis of the western blot results (** *P* < 0.01, *** *P* < 0.001)

To assess whether phosphorylated ERK, MMP-2 and MMP-9 changes were caused by a decrease in PD-L1, we used siRNAs targeting PD-L1 to inhibit the PD-L1 expression. Compared with NC cells, the expression of PD-L1 (Fig. 3a), phosphorylated ERK (Fig. 3b), MMP-2 (Fig. 3c) and MMP-9 (Fig. 3d) was significantly decreased in cells transfected with siRNAs to PD-L1, and confirmed by the cellular immunofluorescence analysis. Western blot detection results were the same as the above (Fig. 3e). These results indicate that the PD-1/PD-L1 signaling pathway is involved in the regulation of MMP-2 and MMP-9 expression and secretion in HTR-8/SVneo cells. The mitogen-activated protein kinase (MAPK) cascade is an important pathway regulating MMP-2 or MMP-9 expression and can respond to extracellular stimuli. Preferentially activates the extracellular signal-regulated kinase-1/2 (ERK1/2) signaling pathway. In HTR-8/SVneo cells transfected with PD-L1 siRNA, ERK1 phosphorylation at 44 kDa (Thr202/Tyr204) increased significantly. However, there was no significant change in total ERK. These results indicate that the in vitro invasion of embryonic trophoblast cells may be mediated by MMP-2 and MMP-9 expression regulated by the MAPK/ERK cascade.

### Silencing of PD-L1 decresed the invasion ability of HTR-8/SVneo cells

**Fig. 4:**
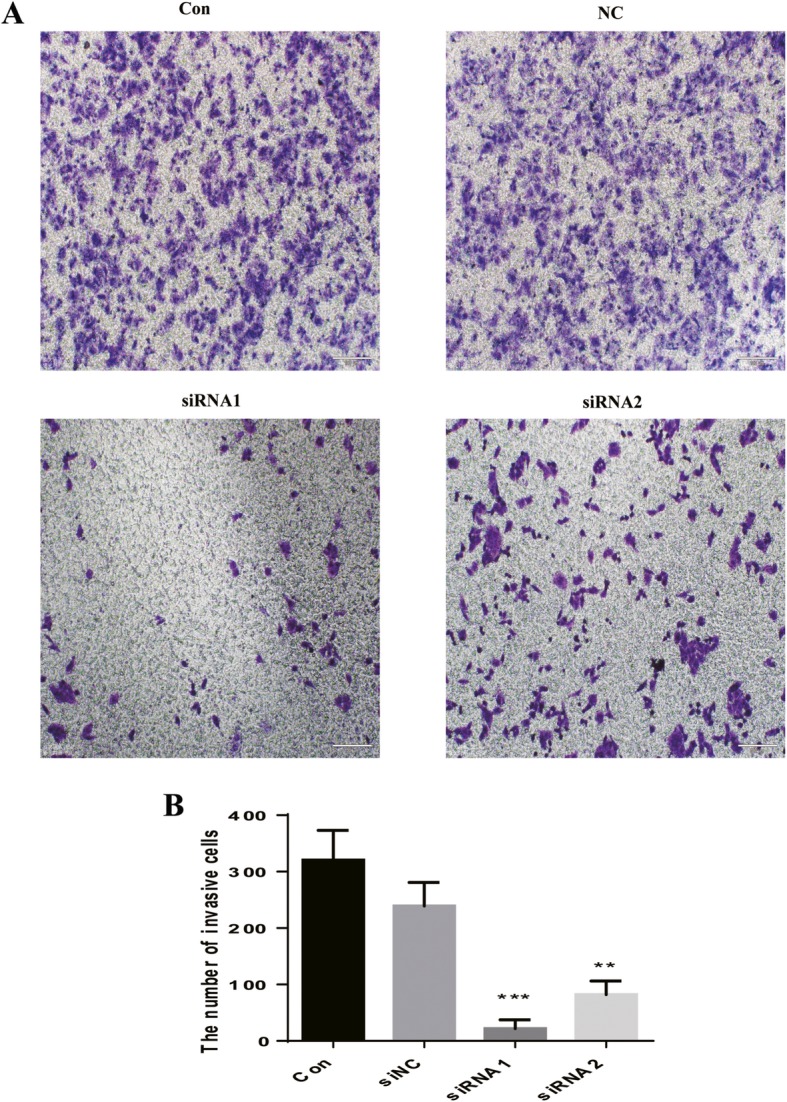
Effect of PD-L1 knockdown on cell invasion in HTR-8/SVneo cells. Parental or HTR-8/SVneo cells transfected with negative control siRNA (NC) or siRNAs (siRNA1 siRNA2) targeting PD-L1 for 48 h were seeded in modified transwell chamber with Matrigel-coated membrane, and after another 24 h, invasive cells that moved through the Matrigel membrane were stained and counted under a microscope (× 100). **a** Showed the nuclei of the invasive cells. **b** Showed statistical analysis of the number of invasive cells. Data was shown as means ± SD from five fields. **P < 0.01 versus the NC group

To evaluate the biological role of PD-L1 in the trophoblast invasion of the placenta, HTR-8/SVneo cells were transfected with PD-L1 siRNAs (siRNA1 and siRNA2) to knock down PD-L1 for use in subsequent studies and used for subsequent studies. PD-L1 expression was significantly reduced in HTR-8/SVneo cells transfected with siRNAs targeting PD-L1, as confirmed by cellular immunofluorescence analysis (Fig. 2a) and Western blot analysis (Fig. 2e). To investigate the effect of PD-L1 on HTR-8/SVneo cell invasion, a Transwell chamber with a Matrigel-coated filter was used. Then 200 ml different HTR-8/SVneo cells suspension were added into the upper chamber. MEM with 15% FBS was added into the lower chamber. Following incubation for 36 h, the invasive cells were stained with purple crystal and recorded under microscope. We found that compared with the NC group cells, HTR-8/SVneo cells transfected with siRNAs targeting PD-L1 had reduced invasion ability (Fig. 4).

## Discussion

The mechanism of abortion caused by AIT has not been unanimously determined. So far, whether the PD-L1 expression in placental trophoblast cells causes the invasion function of trophoblast cells is unknown. In recent years, immune checkpoints have played an important role in the process of cancer research. Among them, PD-1 is one of the most characteristic checkpoint proteins. PD-1 and its ligand PD-L1 are key regulators of T cell immune response and peripheral tolerance induction [21, 22]. The interaction between PD-1 and PD-L1 can result in inhibition of T cells, allowing PD-L1 expressing cancer cells to evade PD-1 positive immune cells [23]. Evading immune surveillance and preventing subsequent rejection of the host immune system are the basis for maintaining uterine embryo and fetal development as they express allogeneic paternal antigens [24]. At the maternal-fetal interface, PD-1 is expressed on activated decidual T cells [25], and during pregnancy, PD-L1 is expressed in embryonic trophoblast cells [26]. In non-gestational endometrium, the density of PD-1^+^/CD3^+^ lymphocytes was lower than that in the first trimester placental sites, suggesting that PD-L1 expressing trophoblast may exploit PD-1/PD-L1 mediated immune suppression in normal gestation. In fact, it has been reported that pregnant mice treated with anti-PD-L1 blocking antibody lose their embryos [27] and a deficiency in PD-L1 has been associated with an increased frequency of fetal resorption and decreased fetal survival [28]. Survival of the developing embryo and fetus requires immune tolerance by inactivating the maternal immune system at the placental-maternal interface, which is thought to be accomplished by trophoblast [24]. Invasion of extravillous trophoblasts plays an important role in embryo implantation and placental formation. If this invasion is inhibited, it will cause abortion due to placental dysfunction.

In this study, we have confirmed that PD-L1 levels are significantly reduced in placental trophoblast cells of pregnant mice with autoimmune thyroiditis. To verify whether the inhibition of the trophoblast cells was affected by inhibition of the PD-L1/PD-L1 signaling pathway, we downregulated PD-L1 expression in HTR-8/SVneo cells, which indicates that abnormally low expression of PD-L1 in HTR-8/SVneo cells results in a significant decrease in the invasive ability of the cells.

The invasion of trophoblast cells is a very complex process that may be affected by many molecules that can affect cell growth, adhesion, differentiation, and degradation of extracellular matrix (ECM), among which, the proteolytic degradation of ECM plays an important role in the process of trophoblast invasion into the endometrium [29]. Matrix metalloproteinases (MMPs) are a key family of proteolytic enzymes involved in trophoblast invasion. Studies have shown that MMP-2 is one of the key enzymes for the degradation of type IV collagen during cell invasion, MMP-9 is identified as trophoblast derived MMP [30]. To investigate how the PD-1/PD-L1 signaling pathway affects cell invasion, we examined the expression levels of MMP-2 and MMP-9 in PD-L1 siRNA-transfected cells. The results showed that the expression of MMP-2 and MMP-9 was down-regulated in cells knocked down by PD-L1. This suggests that attenuation of the PD-1/PD-L1 signaling pathway in the placenta of pregnant mice with AIT may inhibit trophoblast invasion by decreasing the expression of MMP-2 and MMP-9. Previous studies have demonstrated that MMP activation is caused by ERK1/2 phosphorylation [31]. Therefore, we examined the expression of phosphorylated ERK1/2 and total ERK1/2. The results showed that there was no difference in ERK1/2 protein levels in the HTR-8/SVneo cells transfected with PD-L1 siRNA compared with the NC group, but the phosphorylated ERK1/2 levels decreased significantly, indicating that the weakening of the PD-1/PD-L1 signaling pathway leading to phosphorylation of ERK1/2 is reduced. In addition, the results also showed that the expression of MMP-2 and MMP-9 was decreased in HTR-8/SVneo cells transfected with PD-L1 siRNA. Thus, we infer that, in the placenta of pregnant mice with autoimmune thyroiditis, attenuation of the PD-1/PD-L1 signaling pathway may inhibit p-ERK1/2 signaling and its downstream effects on MMP-2 and MMP-9.

In summary, our results suggest that decreased invasiveness of embryonic trophoblasts in autoimmune thyroiditis is associated with down-regulation of PD-1/PD-L1 signaling pathway and inhibition of MMP-2 and MMP-9 expression. However, studies of mouse placenta and in vitro human trophoblast cells do not fully mimic the normal placental uterus. Therefore, the exploration of these findings requires further research.

## Data Availability

All data generated or analysed during this study are included in this published article.

## Abbreviations

AIT: Autoimmune thyroiditis
CTB: Cytotrophoblastic
ERK: Extracellular signal-regulated kinase
EVT: Extravillous trophoblast cells
FBS: Fetal bovine serum
HE: Hematoxylin-eosin
MAPK: Mitogen-activated protein kinase
MMP: Matrix metalloproteinases
PD-1: Programmed cell death-1
PD-L1: Programmed cell death -ligand 1
RPMI: Roswell Park Memorial Institute
